# Preclinical Evaluation of ^177^Lu-rhPSMA-10.1, a Radiopharmaceutical for Prostate Cancer: Biodistribution and Therapeutic Efficacy

**DOI:** 10.2967/jnumed.124.268508

**Published:** 2025-04

**Authors:** Caroline Foxton, Bradley Waldron, Rikke Veggerby Grønlund, Jaime (Jim) Simón, Bart Cornelissen, Edward O’Neill, Daniel Stevens

**Affiliations:** 1 Blue Earth Diagnostics Ltd., Oxford, United Kingdom;; 2 Blue Earth Therapeutics Ltd., Oxford, United Kingdom;; 3 Minerva Imaging, Ølstykke, Denmark;; 4 IsoTherapeutics Group LLC, Angleton, Texas; and; 5 Department of Oncology, University of Oxford, Oxford, United Kingdom

**Keywords:** preclinical, prostate cancer, PSMA, radiopharmaceutical, biodistribution

## Abstract

^177^Lu-rhPSMA-10.1 is a novel radiohybrid prostate-specific membrane antigen (PSMA)–targeted radiopharmaceutical therapy for prostate cancer. We conducted preclinical analyses on non–tumor-bearing BALB/c mice and on prostate cancer human xenograft mouse models (LNCAP and 22Rv1 xenografts) to evaluate its biodistribution and therapeutic efficacy. **Methods:** Longitudinal biodistribution of ^177^Lu-rhPSMA-10.1 was evaluated in BALB/c mice and 22Rv1 xenografts. Tissues of interest were harvested, and radioactivity was measured 1–168 h after injection of 1 MBq of ^177^Lu-rhPSMA-10.1 (4 per time point). Longitudinal biodistribution was compared with ^177^Lu-PSMA-I&T (1 MBq) in BALB/c mice and at a single time point (15 h) in 22Rv1 xenografts. The therapeutic efficacy of a single administration of 15, 30, or 45 MBq of ^177^Lu-rhPSMA-10.1 in LNCaP xenografts and 30 MBq of ^177^Lu-rhPSMA-10.1, ^177^Lu-PSMA-617, or ^177^Lu-PSMA-I&T in 22Rv1 xenografts (8 per group) was evaluated. Efficacy versus vehicle was evaluated on the basis of relative tumor volume and survival up to 49 d after administration. Statistical significance was evaluated with *t* testing (biodistribution data), 2-way repeated-measures ANOVA (tumor volume [analyzed until 3 per group remained]), or Kaplan–Meier log-rank analyses (survival). **Results:** Biodistribution of ^177^Lu-rhPSMA-10.1 in the BALB/c and 22Rv1 xenografts showed rapid clearance from blood and other normal tissues within 48 h, with the kidney showing the highest normal-organ uptake. Kidney uptake and retention were lower for ^177^Lu-rhPSMA-10.1 than for ^177^Lu-PSMA-I&T (6.5-fold lower at 12 h in BALB/c mice and 6.4-fold lower at 15 h in 22Rv1 xenografts; *P* < 0.01). High and sustained ^177^Lu-rhPSMA-10.1 tumor uptake was observed in 22Rv1 xenografts. This uptake was 2.3-fold higher than that of ^177^Lu-PSMA-I&T (15 h; *P* < 0.05). When efficacy was evaluated, ^177^Lu-rhPSMA-10.1 significantly suppressed tumor growth versus vehicle from day 11 (*P* < 0.05) in LNCaP xenografts in a dose-dependent manner and from day 18 (*P* < 0.05) in 22Rv1 xenografts and significantly prolonged median survival versus vehicle in both models. In 22Rv1 xenografts, ^177^Lu-rhPSMA-10.1 suppressed tumor growth versus vehicle to a greater extent than did ^177^Lu-PSMA-I&T (significant growth inhibition from day 25 [*P* < 0.05]) and similarly in extent to ^177^Lu-PSMA-617 (from day 18 [*P* < 0.05]). Overall, compared with ^177^Lu-PSMA-I&T, ^177^Lu-rhPSMA-10.1 suppressed tumor growth for longer than ^177^Lu-PSMA-617 (inhibition from day 39 onward [*P* < 0.05] versus on day 49 only [*P* < 0.05]). **Conclusion:** In preclinical models, ^177^Lu-rhPSMA-10.1 shows a favorable tumor-to-kidney uptake ratio, and significant antitumor effects, indicating it to be a promising next-generation radiopharmaceutical therapy.

## Visual Abstract

**Figure:**
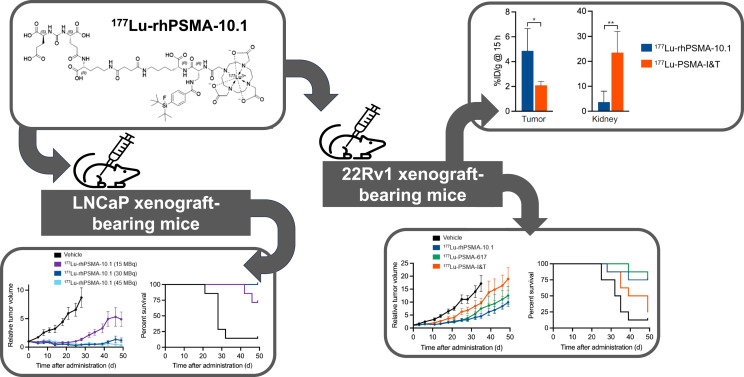

The recent development of ^177^Lu-labeled prostate-specific membrane antigen (PSMA)–targeted radiopharmaceutical therapy (RPT) has provided further therapeutic options for patients with metastatic castration-resistant prostate cancer (mCRPC) who show disease progression after conventional management with androgen axis drugs and chemotherapy (1–4).

The first U.S. Food and Drug Administration–approved PSMA-targeted RPT for prostate cancer (PCa), ^177^Lu-PSMA-617 (Pluvicto; Novartis), has been shown to extend progression-free survival in men with mCRPC before and after taxane chemotherapy and to extend overall survival in posttaxane mCRPC (3). A further similar investigational RPT, ^177^Lu-PSMA-I&T, has been shown to extend progression-free survival in the pretaxane mCRPC setting, with acceptable tolerability (1*,*^5^).

However, despite these advances, there remains a need for next-generation RPT agents that address the lack of efficacy experienced by approximately one third of patients undergoing treatment with the current agents and to lessen the potential impact of radiation-induced nephropathy (6*,*^7^).

^177^Lu-PSMA-617 data from prospective clinical trials suggest that providing a greater absorbed radiation dose to the tumor may achieve improved outcomes, such as a greater PSA response (8*,*^9^). However, limiting the dose to the kidneys is also of prime clinical consideration since the kidneys are a dose-limiting organ (10). In patients with late-stage disease and limited life expectancy, the risk of delayed radiation nephropathy from multiple cycles of RPT is less likely to impact clinical decision making. However, as PSMA-targeted RPT moves earlier in the treatment paradigm, balancing the benefit and risk of radiation exposure becomes more critical. Multiple ongoing studies such as PSMAddition (NCT04720157) and the Nautilus Trial (NCT06066437) are evaluating RPT earlier in the disease continuum in patients with longer life expectancy, and long-term follow-up from these patients is awaited. Ultimately, the balance of radiation dose to the tumor versus the kidneys will likely become the key gating factor in achievable clinical outcomes.

A novel radiohybrid (rh) technology platform has enabled engineering of PSMA ligands that can be labeled with ^18^F for diagnostic imaging or with α- or β-emitting radiometals for RPT (11). A diagnostic PET radiopharmaceutical, ^18^F-flotufolastat (^18^F-rhPSMA-7.3), developed using this platform was recently approved by the Food and Drug Administration for PSMA PET in men with PCa (12–14). In addition, preliminary assessments have identified a ^177^Lu-labeled rhPSMA, ^177^Lu-rhPSMA-10.1 (Supplemental Fig. 1; supplemental materials are available at http://jnm.snmjournals.org), as a lead candidate for RPT on the basis of its low uptake in the kidneys, rapid blood clearance, and high accumulation in tumors (15*,*^16^).

Here, we present the findings of a comprehensive series of preclinical biodistribution and efficacy studies on healthy animals and PCa human xenograft mouse models to evaluate the therapeutic potential of the RPT agent ^177^Lu-rhPSMA-10.1.

## MATERIALS AND METHODS

### Cell Lines

Two PSMA-expressing PCa cell lines were used to simulate different stages of PCa. The LNCaP (lymph node carcinoma of the prostate) cell line is an androgen-dependent model with high levels of PSMA expression, whereas the 22Rv1 line is androgen-independent with moderate or heterogeneous PSMA expression (17).

The 22Rv1 cell line was sourced from the American Type Culture Collection (CRL-2505) and from Sigma-Aldrich. The LNCaP cell line was sourced from the American Type Culture Collection (CRL-1740). PSMA expression was confirmed by flow cytometry. All cell lines were maintained in complete Roswell Park Memorial Institute medium (10% fetal bovine serum, l-glutamine, penicillin/streptomycin). All cell lines were tested for *Mycoplasma* contamination, and its absence was confirmed before all experiments.

### Animals and Tumor Xenograft Models

All animal experiments were conducted in accordance with animal welfare regulations, including the relevant Institutional Animal Care and Use Committee in the United States, the National Animal Experiments Inspectorate under the Ministry of Environment and Food of Denmark, and the U.K. Animals (Scientific Procedures) Act of 1986, with local ethical committee approval. All animals were group-housed and allowed an acclimatization period of 7–8 d before initiation of the experimental procedures.

Studies on healthy animals were performed on BALB/c mice (supplied by Envigo).

For the biodistribution studies, the 22Rv1 cells were grown as a xenograft in male C.B-17/lcr severe combined immunodeficient (SCID) mice (supplied by Charles River Laboratories) through subcutaneous injection in the right flank (1 × 10^6^ cells in 100 μL of 1:1 phosphate-buffered saline:Matrigel [Corning]). A 2-stage study was conducted, with the 22Rv1 xenograft–bearing mice in the first stage being inoculated at 6 wk old and those in the second stage at 14 wk.

For efficacy studies, 22Rv1 or LNCaP cells were grown as xenografts in male Naval Medical Research Institute (NMRI) nude mice (supplied by Janvier Labs) through subcutaneous injection in the right flank when the animals were 7–8 wk old (3 × 10^6^ or 5 × 10^6^ cells in 100 μL of 1:1 phosphate buffered saline:Matrigel for 22Rv1 or LNCaP, respectively).

When tumors were of sufficient size (mean, 100–300 mm^3^), animals were selected for inclusion. Animals were killed if tumor volume reached 1,500 mm^3^; if they had a tumor burden of at least 10% of their body weight; a body weight loss or gain of 15% or 20%, respectively; or signs of tumor ulceration, distress, or impaired movement due to tumors.

### Radiolabeling Procedures

The rhPSMA-10.1 precursor was sourced from Almac Sciences, the PSMA-617 precursor from MedChemExpress, and the PSMA-I&T precursor from Huayi Isotopes (for BALB/c studies) and ABX (for 22Rv1 biodistribution studies). All radiopharmaceuticals were radiolabeled, formulated, and quality-controlled using optimized versions of previously described methods (15). For the BALB/c biodistribution study, ^177^Lu-rhPSMA-10.1 was produced at a molar activity of 25 MBq/nmol, and for the 22Rv1 biodistribution study, ^177^Lu-rhPSMA-10.1 was produced at a molar activity of 25 MBq/nmol and ^177^Lu-PSMA-I&T at 42 MBq/nmol. For all efficacy studies, all radiopharmaceuticals were produced at a molar activity of 60 MBq/nmol.

### Biodistribution Studies

To assess the biodistribution of ^177^Lu-rhPSMA-10.1 in healthy mice, BALB/c mice received a single bolus injection of 1 MBq of either ^177^Lu-rhPSMA-10.1 or ^177^Lu-PSMA-I&T into the tail vein (4 per time point). Tissues of interest were harvested for radioactivity measurement at 1–168 h after injection.

The 22Rv1 xenograft–bearing SCID mice were used to examine the biodistribution of ^177^Lu-rhPSMA-10.1 in a further longitudinal biodistribution study. ^177^Lu-rhPSMA-10.1 (1 MBq) was administered intravenously to 22Rv1 tumor–bearing mice (4 per time point) 1–168 h before tissue harvesting for radioactivity measurement. The study was conducted in a 2-stage process, with 2 time points (15 and 24 h) evaluated in the first phase and 4 (1 h, 6 h, 2 d, and 7 d) evaluated in the second phase. For comparison with ^177^Lu-PSMA-I&T at a single time point, 1 MBq of ^177^Lu-PSMA-I&T was administered intravenously to 22Rv1 tumor–bearing mice (*n* = 3), and tissues were harvested for radioactivity measurement 15 h later.

The radioactivity in harvested tissues was measured via ex vivo γ-counting, using a HiDEX automated γ-counter with a built-in sample balance. Radiopharmaceutical uptake was calculated as percentage injected dose per gram of tissue (%ID/g).

### Therapeutic Efficacy Studies

#### Efficacy of ^177^Lu-rhPSMA-10.1 in LNCaP Xenograft–Bearing NMRI Mice (Dose–Response Study)

A single bolus of ^177^Lu-rhPSMA-10.1 (15, 30, or 45 MBq) was administered on day 0 to LNCaP xenograft–bearing mice (8 per group). On the day before dosing, there were no significant differences in tumor volume or body weight between the groups. Tumor volume and body weight were measured on day 0 and assessed twice a week for up to 49 d after ^177^Lu-rhPSMA-10.1 administration. Tumor volume was calculated using 0.52(length × width^2^). Efficacy evaluations were based on relative tumor growth (change from day 0 in tumor volume) and survival of mice treated with radiopharmaceuticals versus vehicle, up to 49 d after treatment.

#### Efficacy of ^177^Lu-rhPSMA-10.1, ^177^Lu-PSMA-617, and ^177^Lu-PSMA-I&T in 22Rv1 Xenograft–Bearing NMRI Mice

To confirm the efficacy of ^177^Lu-rhPSMA-10.1 in another PCa xenograft model, the antitumor activity of ^177^Lu-rhPSMA-10.1 was also examined in 22Rv1 xenograft–bearing mice, with vehicle, ^177^Lu-PSMA-617, and ^177^Lu-PSMA-I&T administered to comparator groups. No significant differences in tumor volume or body weight existed between the groups before dosing. A single bolus of ^177^Lu-rhPSMA-10.1, ^177^Lu-PSMA-617, or ^177^Lu-PSMA-I&T (30 MBq each) was administered to 22Rv1 tumor–bearing mice (8 per group) on day 0 (23 d after tumor cell inoculation). Study drug treatment was masked throughout. Tumor volume was monitored twice a week for 49 d and evaluated as described for the LNCaP xenograft–bearing mice.

### Hematologic Analysis

Hematologic analysis was conducted on blood samples collected from the xenograft-bearing NMRI mice used in the efficacy analysis. Whole blood samples (100 μL) were drawn from the sublingual vein and collected in ethylenediaminetetraacetic acid vials the day before dosing (day −1) and then on days 14 and 28. The evaluated hematologic parameters (as listed in Supplemental Table 1) were measured with the ProCyte Dx Hematology Analyzer (IDEXX) using mouse settings.

### Statistical Analyses

All data are summarized as mean ± SD or SEM. Biodistribution data were analyzed with unpaired *t* tests. For analysis of tumor volume, data were analyzed up until the time point when 3 per group remained using a 2-way repeated-measures ANOVA, with the Tukey honest significant difference test used to control the familywise error rate. For the ^177^Lu-PSMA-I&T 30-MBq group, there was 1 time point at which data were missing (assumed at random), and a mixed-effects model was used. For the survival analysis (time-to-event data), Kaplan–Meier survival analysis was performed, with log-rank tests used to evaluate statistical significance between groups (*P* ≤ 0.05). All statistical analyses were performed using Prism (version 9.5.1; GraphPad Software, Inc.).

## RESULTS

### Biodistribution of ^177^Lu-rhPSMA-10.1

**FIGURE 1.**
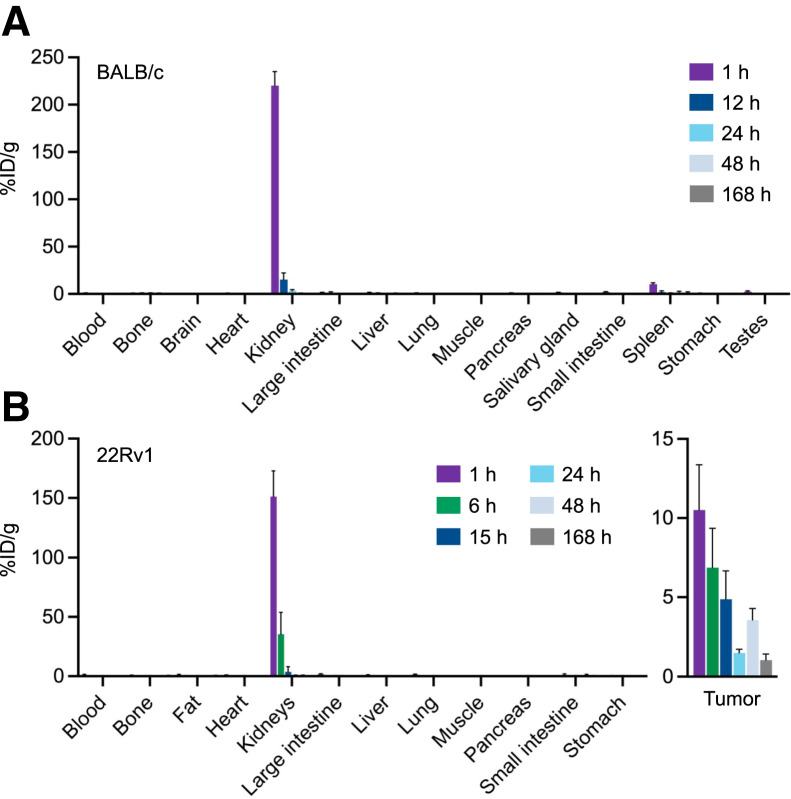
Longitudinal biodistribution of ^177^Lu-rhPSMA-10.1 in non–tumor-bearing BALB/c mice (A) and 22Rv1 xenograft–bearing SCID mice (B).

The longitudinal biodistribution analysis of ^177^Lu-rhPSMA-10.1 in non–tumor-bearing BALB/c mice showed the normal organ with the highest uptake to be the kidney (Fig. 1A; Supplemental Fig. 2). The high kidney uptake observed at 1 h (220 %ID/g) cleared rapidly such that activity was negligible by 48 h after injection. Transient uptake was observed in the spleen at the 1-h time point (10.4 %ID/g) and had cleared almost completely by 24 h. No other organ (including the brain) showed any significant uptake.

The longitudinal biodistribution of ^177^Lu-rhPSMA-10.1 was also examined in a PSMA-expressing PCa xenograft model, 22Rv1. High and sustained uptake was observed in the tumors (10.5 %ID/g at the 1-h time point), and the highest normal-organ uptake, which cleared rapidly, was in the kidney (151 %ID/g at the 1-h time point), with no other organ showing notable uptake (Fig. 1B).

### Biodistribution of ^177^Lu-rhPSMA-10.1 Compared with ^177^Lu-PSMA-I&T

**FIGURE 2.**
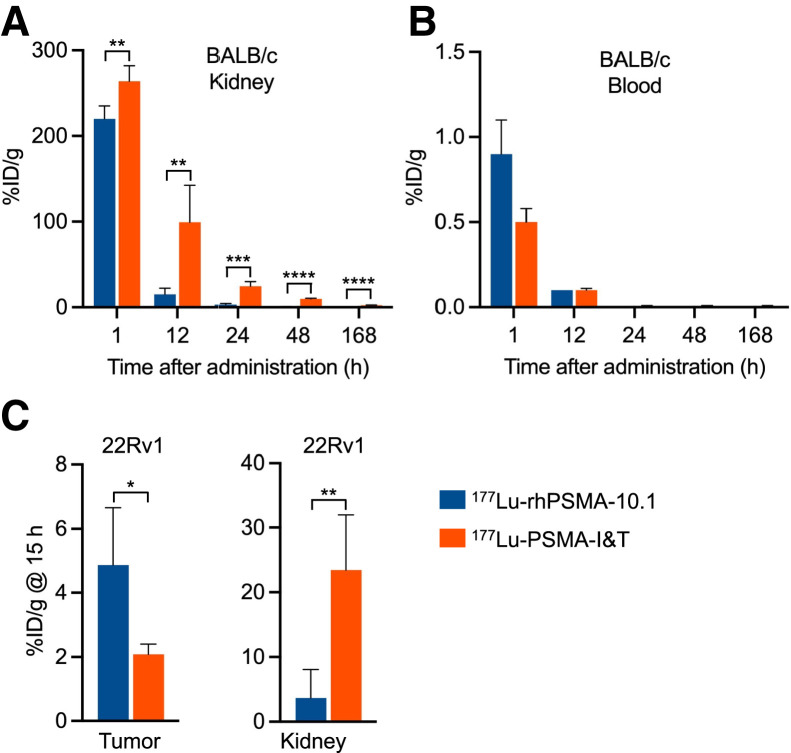
(A) Kidney uptake of ^177^Lu-rhPSMA-10.1 and ^177^Lu-PSMA-I&T in BALB/c mice. (B) Blood clearance of ^177^Lu-rhPSMA-10.1 and ^177^Lu-PSMA-I&T in BALB/c mice. (C) Tumor and kidney uptake of ^177^Lu-rhPSMA-10.1 and ^177^Lu-PSMA-I&T in 22Rv1 xenograft–bearing SCID mice at single time point. *P ≤ 0.05. **P ≤ 0.01. ***P ≤ 0.001.

The longitudinal biodistribution of ^177^Lu-rhPSMA-10.1 was also compared with that of the reference radiopharmaceutical ^177^Lu-PSMA-I&T in BALB/c mice. Kidney uptake and retention were significantly lower for ^177^Lu-rhPSMA-10.1 than for ^177^Lu-PSMA-I&T at all evaluated time points (*P* < 0.01), with 6.5-fold lower levels of ^177^Lu-rhPSMA-10.1 than of ^177^Lu-PSMA-I&T observed at 12 h (15.2 %ID/g vs. 99.3 %ID/g, respectively [*P* < 0.01]; Fig. 2A). Both ^177^Lu-rhPSMA-10.1 and ^177^Lu-PSMA-I&T were rapidly cleared from the blood (Fig. 2B). As with ^177^Lu-rhPSMA-10.1, transient spleen uptake was noted for ^177^Lu-PSMA-I&T at the 1-h time point and had mostly cleared by 24 h. At 1 h, spleen uptake was 16.3 %ID/g for ^177^Lu-PSMA-I&T versus 10.4 %ID/g for ^177^Lu-rhPSMA-10.1. No other organ showed notable uptake of ^177^Lu-rhPSMA-10.1 or ^177^Lu-PSMA-I&T (Supplemental Fig. 2).

Biodistribution of ^177^Lu-rhPSMA-10.1 and ^177^Lu-PSMA-I&T was also compared at a single time point in 22Rv1 tumor–bearing SCID mice to evaluate their corresponding tumor-to-kidney uptake ratios. As shown in Figure 2C, at 15 h after injection ^177^Lu-rhPSMA-10.1 had significantly lower kidney uptake than did ^177^Lu-PSMA-I&T (6.4-fold, *P* < 0.01; 3.7 %ID/g vs. 23.5 %ID/g, respectively), whereas tumor uptake was significantly higher with ^177^Lu-rhPSMA-10.1 than with ^177^Lu-PSMA-I&T (2.3-fold, *P* < 0.05; 4.9 %ID/g vs. 2.1 %ID/g, respectively), resulting in a more favorable tumor-to-kidney ratio for ^177^Lu-rhPSMA-10.1 (2.3 ± 1.14 vs. 0.1 ± 0.03). No other organ showed notable uptake of ^177^Lu-rhPSMA-10.1 or ^177^Lu-PSMA-I&T (Supplemental Fig. 3).

### Antitumor Efficacy of ^177^Lu-rhPSMA-10.1 in PCa Xenograft Models

#### Dose–Response Relationship of ^177^Lu-rhPSMA-10.1 in LNCaP Xenograft–Bearing NMRI Mice

**FIGURE 3.**
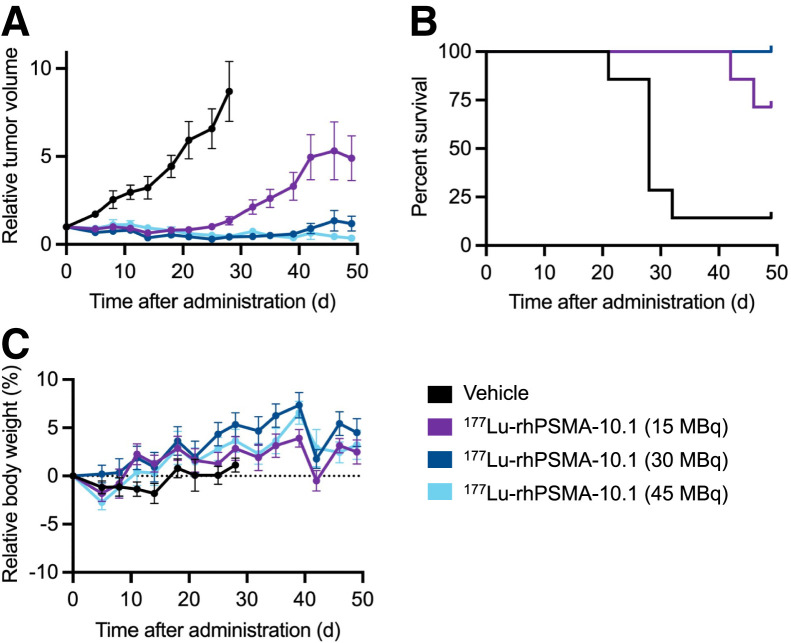
Therapeutic efficacy and tolerability of single administration of ^177^Lu-rhPSMA-10.1 (15, 30, and 45 MBq) in LNCaP tumor–bearing mice: relative tumor volume (A), survival (B), and relative body weight (C).

At all evaluated doses (15, 30, and 45 MBq), ^177^Lu-rhPSMA-10.1 significantly suppressed tumor growth from day 11 (*P* < 0.05) to day 28 (*P* < 0.0001; Fig. 3A) versus vehicle. Furthermore, tumor growth was significantly reduced with 30 and 45 MBq compared with 15 MBq from day 35 (*P* < 0.01) to day 49 (*P* < 0.0001; Fig. 3A), indicative of a dose–response relationship.

Median survival of mice was 28 d in the vehicle group and was not reached for any of the ^177^Lu-rhPSMA-10.1 groups at the study end, with all animals still alive in the 30- and 45-MBq groups on day 49 (Fig. 3B). A significant difference in survival was observed between all ^177^Lu-rhPSMA-10.1 doses and vehicle (*P* < 0.01, < 0.001, and < 0.01 for 15-, 30-, and 45-MBq doses, respectively [log-rank Mantel–Cox]).

^177^Lu-rhPSMA-10.1 was well tolerated in the LNCaP xenograft–bearing mice, and no significant weight loss was noted in any of the treatment groups throughout the study (Fig. 3C).

#### Efficacy of ^177^Lu-rhPSMA-10.1, ^177^Lu-PSMA-617, and ^177^Lu-PSMA-I&T in 22Rv1 Xenograft–Bearing NMRI Mice

**FIGURE 4.**
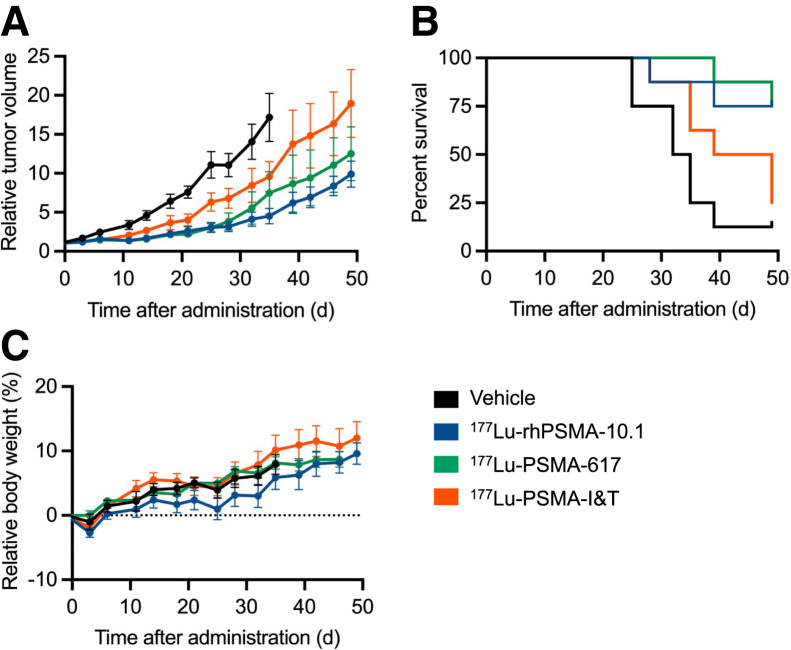
Therapeutic efficacy and tolerability of single administration (30 MBq) of ^177^Lu-rhPSMA-10.1 in 22Rv1 tumor–bearing mice, compared with ^177^Lu-PSMA-617 and ^177^Lu-PSMA-I&T: relative tumor volume (A), survival (B), and relative body weight (C).

In the 22Rv1 xenograft model, ^177^Lu-rhPSMA-10.1 significantly suppressed tumor growth from day 18 (*P* < 0.05) to day 35 (*P* < 0.0001) versus vehicle (Fig. 4A).

Significant suppression of tumor growth compared with vehicle was also observed with ^177^Lu-PSMA-617 from day 18 (*P* < 0.05) to day 35 (*P* < 0.0001), and to a lesser extent with ^177^Lu-PSMA-I&T, which was shown to inhibit growth from day 25 (*P* < 0.05) to day 35 (*P* < 0.0001; Fig. 4A).

Compared with ^177^Lu-PSMA-I&T, ^177^Lu-rhPSMA-10.1 significantly reduced tumor growth from day 39 (*P* < 0.05) to day 49 (*P* < 0.01), whereas ^177^Lu-PSMA-617 significantly reduced tumor growth on day 49 only (*P* < 0.05).

Median survival of mice was 33.5 d in the vehicle group and 44 d in the ^177^Lu-PSMA-I&T group and was not reached for the ^177^Lu-rhPSMA-10.1 and ^177^Lu-PSMA-617 groups (Fig. 4B). A significant difference in survival was observed between the vehicle control and either ^177^Lu-rhPSMA-10.1 or ^177^Lu-PSMA-617 (*P* < 0.05 and < 0.01, respectively [log-rank Mantel–Cox]).

As seen in the LNCaP model, all treatments were well tolerated in the 22Rv1 xenograft–bearing mice, with no significant weight loss observed in any of the treatment groups (Fig. 4C). In terms of hematologic assessments, all parameters examined were within accepted reference ranges reported in the literature (Supplemental Table 1) (18*,*^19^).

## DISCUSSION

PSMA-targeted RPT has been shown to increase overall survival compared with standard-of-care treatment in patients with mCRPC (3), but a need remains for next-generation radiopharmaceuticals that can provide improved tumor uptake while minimizing uptake to the kidneys, which remain one of the most significant organs at risk. Ultimately, on the basis of emerging longer-term clinical data, an upper dose limit will likely be applied to the kidney, which will be dose-limiting in terms of the total cumulative radioactivity that can be administered to patients. In this scenario, the RPT that provides the highest achievable tumor-absorbed radiation dose within this predefined acceptable kidney exposure will offer the best outcomes for patients.

To this end, a next-generation RPT, ^177^Lu-rhPSMA-10.1, has been developed with optimized properties for therapeutic use (15*,*^16^). It has been shown to have PSMA-binding affinity and PSMA-mediated internalization in LNCaP cells similarly high to those of ^177^Lu-PSMA-617 and ^177^Lu-PSMA-I&T (with all 3 agents having PSMA-binding affinities in the low-nanomolar range and high internalization rates of >145% of the reference ligand ^125^IBA-KuE) (15). Furthermore, ^177^Lu-rhPSMA-10.1 has a higher binding strength to human serum albumin than either ^177^Lu-PSMA-617 or ^177^Lu-PSMA-I&T, and this higher binding strength is thought to provide an optimal balance between clearance from healthy organs and preservation of high tumor accumulation (15). Here, we conducted a series of preclinical evaluations to fully characterize the biodistribution and to evaluate the therapeutic efficacy of ^177^Lu-rhPSMA-10.1 in 2 PSMA-expressing PCa models.

^177^Lu-rhPSMA-10.1 was well tolerated across all studies, with no significant effect on body weight or hematologic parameters. A similar biodistribution profile was observed in both the non–tumor-bearing BALB/c mice and the 22Rv1 xenograft–bearing mice. The biodistribution was typical of other PSMA-targeted radiolabeled compounds, showing rapid blood clearance, with most activity excreted via the kidneys and urinary bladder but with high levels of tumor retention. As has been shown in PCa xenograft models for other PSMA-targeted radiolabeled compounds, the most significant normal-organ uptake was in the kidneys (20–22). However, we showed that levels of ^177^Lu-rhPSMA-10.1 in the kidney decrease substantially by 24–48 h after administration, with almost complete clearance by 168 h. The kidney uptake concurs with findings of a previous study of ^177^Lu-rhPSMA-10.1 in LNCaP tumor–bearing mice and is a due to specific binding to PSMA receptors in the kidney and to the fact that ^177^Lu-rhPSMA-10.1 is excreted primarily via the renal system (16).

Transient spleen uptake was noted at 1 h and had mostly cleared by 12 h in non–tumor-bearing BALB/c mice, similar to what was reported by Wurzer et al. (16). Several other PSMA-targeted ligands also demonstrated high initial spleen uptake in mouse models—an occurrence that appears to involve both a PSMA-independent and a PSMA-dependent process that can be partially blocked with a competitor such as 2-PMPA (20*,*^21^*,*^23^).

In addition to the kidney, the bone marrow and salivary glands are considered toxicity-limiting organs for RPT (24). Both xenograft models evaluated here showed minimal uptake in bone marrow. Although we evaluated only salivary gland uptake in the BALB/c model, our data showing minimal uptake in salivary glands align with previous data on LNCaP xenograft–bearing mice (16).

A prior single-time-point (24 h) analysis in LNCaP xenograft–bearing mice showed ^177^Lu-rhPSMA-10.1 to have one of the lowest kidney uptakes of several evaluated PSMA-targeted radiopharmaceuticals, including ^177^Lu-PSMA-I&T and ^177^Lu-PSMA-617, while also preserving high tumor uptake (15). Consistent with this, our data also showed ^177^Lu-rhPSMA-10.1 to have a favorable biodistribution profile compared with ^177^Lu-PSMA-I&T, with a 6.5-fold lower kidney uptake at 12 h and a more favorable tumor-to-kidney ratio than ^177^Lu-PSMA-I&T.

Given the encouraging tumor-to-kidney ratio for ^177^Lu-rhPSMA-10.1, the therapeutic efficacy of ^177^Lu-rhPSMA-10.1 was investigated in LNCaP xenograft–bearing mice. Our data showed ^177^Lu-rhPSMA-10.1 to significantly reduce tumor growth in a dose-dependent manner, with even 15 MBq of ^177^Lu-rhPSMA-10.1 achieving significant reductions in tumor growth and prolonged survival compared with vehicle. Furthermore, ^177^Lu-rhPSMA-10.1 significantly suppressed tumor growth compared with vehicle in the lower-PSMA-expressing 22Rv1 model that is more reflective of the human patient. We note that suppression of tumor growth was lower and less sustained in 22Rv1 than in LNCaP xenograft–bearing mice, likely because of the lower levels of PSMA expression. However, in both models, a single cycle of ^177^Lu-rhPSMA-10.1 suppressed tumor growth for longer than 4 wk. In clinical practice, it is likely that repeated cycles of treatment would occur, leading to even longer tumor suppression.

When compared with other ^177^Lu-labeled PSMA ligands, ^177^Lu-rhPSMA-10.1 achieved significantly greater tumor growth suppression than did ^177^Lu-PSMA-I&T, consistent with our biodistribution data showing higher uptake and retention in tumors for ^177^Lu-rhPSMA-10.1 than for ^177^Lu-PSMA-I&T. ^177^Lu-PSMA-617 demonstrated comparable tumor suppression to ^177^Lu-rhPSMA-10.1 until the later time points, whereupon ^177^Lu-rhPSMA-10.1 showed longer-lasting growth suppression. When efficacy was compared with that of ^177^Lu-PSMA-I&T, ^177^Lu-rhPSMA-10.1 significantly reduced tumor growth from days 32 to 49, whereas ^177^Lu-PSMA-617 significantly reduced tumor growth on day 49 only. This may reflect the improved tumor uptake or retention of ^177^Lu-rhPSMA-10.1 compared with ^177^Lu-PSMA-617 (15).

The favorable tumor-to-kidney ratio and antitumor efficacy of ^177^Lu-rhPSMA-10.1 shown here in preclinical models have recently been demonstrated among the first patients with PCa to receive ^177^Lu-rhPSMA-10.1 as part of an early clinical experience in Germany (25*,*^26^). Rinscheid et al. showed—in an intrapatient comparative dosimetry study of ^177^Lu-rhPSMA-10.1 and ^177^Lu-PSMA-I&T in 4 patients with mCRPC—that ^177^Lu-rhPSMA-10.1 has a significantly increased tumor-absorbed dose, resulting in an improved tumor-to-kidney therapeutic index compared with ^177^Lu-PSMA-I&T (26). Moreover, encouraging efficacy and survival data were shown among the same 4 patients, who all demonstrated a PSA improvement ranging from 35% to 100%, with 3 patients achieving more than 80% reductions (25).

A particular strength of our work is the evaluation of ^177^Lu-rhPSMA-10.1 in multiple preclinical models. Along with existing data (15), this facilitates understanding of the biodistribution of ^177^Lu-rhPSMA-10.1 in healthy animals and at distinct disease stages. Furthermore, to understand efficacy at different stages, we conducted evaluations on both 22Rv1 and LNCaP xenografts. The androgen-dependent, highly PSMA-expressing LNCaP cell line is derived from metastatic deposits and provides a model of advanced disease, whereas the androgen-independent 22Rv1 line has a more moderate PSMA expression that is more reflective of the heterogeneous PSMA expression in humans (17). There are, however, some limitations to our work. Although 22Rv1 xenografts were used for both the biodistribution and the efficacy studies, different mouse strains (SCID and NMRI, respectively) were used as hosts. This likely had little impact on the reported findings, since all comparisons were made within groups of xenografts in the same strain. Also, whereas each of the xenografts used in this study may have had heterogeneous tumor growth rates, we selected only animals with tumors of uniform size for inclusion. Further limitations include that we evaluated only salivary gland uptake in the BALB/c model. Future clinical studies are needed to explore ^177^Lu-rhPSMA-10.1 salivary gland uptake in more detail. Finally, it is pertinent to note that kidney uptake of radiopharmaceuticals does not necessarily translate between mice and humans. For instance, our data, and those of other preclinical studies (21*,*^22^), have shown significant uptake for ^177^Lu-PSMA-I&T in the kidneys. Although dosimetry studies on humans have also shown a slightly higher kidney uptake for ^177^Lu-PSMA-I&T than for ^177^Lu-PSMA-617, the difference is not considered clinically significant (27*,*^28^). However, as indicated by the data reported above from a compassionate-use program in Germany, the favorable safety profile, tumor-to-kidney ratio, and antitumor effects demonstrated here for ^177^Lu-rhPSMA-10.1 in preclinical models were also noted among the first patients to receive ^177^Lu-rhPSMA-10.1, and so the results of several ongoing clinical trials of ^177^Lu-rhPSMA-10.1 in patients across the PCa disease spectrum (NCT05413850, NCT06066437, and NCT06105918) are eagerly anticipated.

## CONCLUSION

These preclinical analyses demonstrate a favorable biodistribution profile for ^177^Lu-rhPSMA-10.1, with high tumor accumulation and low kidney uptake in preclinical models. ^177^Lu-rhPSMA-10.1 also demonstrated significant therapeutic efficacy in 2 PCa human xenograft mouse models that compared favorably with that of ^177^Lu-PSMA-617 and ^177^Lu-PSMA-I&T.

## DISCLOSURE

This work was funded by Blue Earth Therapeutics Ltd. Caroline Foxton is an employee of Blue Earth Diagnostics Ltd. Bradley Waldron and Daniel Stevens are employees of Blue Earth Therapeutics Ltd. Rikke Veggerby Grønlund is an employee of Minerva Imaging. Jaime (Jim) Simón is an employee of IsoTherapeutics Group LLC. No other potential conflict of interest relevant to this article was reported.

## Notes

KEY POINTS **QUESTION:** Does ^177^Lu-rhPSMA-10.1 offer appropriate preclinical biodistribution and antitumor efficacy for RPT of PCa? **PERTINENT FINDINGS:** In preclinical evaluations, ^177^Lu-rhPSMA-10.1 showed a favorable biodistribution with fast clearance from healthy tissues while preserving high levels of tumor uptake. **IMPLICATIONS FOR PATIENT CARE:** Preclinical data indicate a more favorable tumor-to-kidney uptake for ^177^Lu-rhPSMA-10.1 than for ^177^Lu-PSMA-I&T and a greater tumor suppression, suggesting that it is a promising next-generation RPT.
